# Inhibition of Galectins and the P2X7 Purinergic Receptor as a Therapeutic Approach in the Neurovascular Inflammation of Diabetic Retinopathy

**DOI:** 10.3390/ijms24119721

**Published:** 2023-06-03

**Authors:** Caterina Claudia Lepre, Marina Russo, Maria Consiglia Trotta, Francesco Petrillo, Fabiana Anna D’Agostino, Gennaro Gaudino, Giovanbattista D’Amico, Maria Rosaria Campitiello, Erminia Crisci, Maddalena Nicoletti, Carlo Gesualdo, Francesca Simonelli, Michele D’Amico, Anca Hermenean, Settimio Rossi

**Affiliations:** 1 “Aurel Ardelean” Institute of Life Sciences, Vasile Goldis Western University of Arad, 310144 Arad, Romania; 2 Department of Experimental Medicine, University of Campania “Luigi Vanvitelli”, 80138 Naples, Italy; 3 Ph.D. Course in Translational Medicine, Department of Experimental Medicine, University of Campania “Luigi Vanvitelli”, 80138 Naples, Italy; 4 Multidisciplinary Department of Medical, Surgical and Dental Sciences, University of Campania “Luigi Vanvitelli”, 80138 Naples, Italy; 5 School of Anesthesia and Intensive Care, University of Foggia, 71122 Foggia, Italy; 6 School of Geriatrics, University of Studies of L’Aquila, 67010 L’Aquila, Italy; 7 Department of Obstetrics and Gynecology and Physiopathology of Human Reproduction, ASL Salerno, 84124 Salerno, Italy

**Keywords:** diabetic retinopathy, neuroinflammation, ROS, ER stress, NLRP3 inflammasome, galectins, P2X7R

## Abstract

Diabetic retinopathy (DR) is the most frequent microvascular retinal complication of diabetic patients, contributing to loss of vision. Recently, retinal neuroinflammation and neurodegeneration have emerged as key players in DR progression, and therefore, this review examines the neuroinflammatory molecular basis of DR. We focus on four important aspects of retinal neuroinflammation: (i) the exacerbation of endoplasmic reticulum (ER) stress; (ii) the activation of the NLRP3 inflammasome; (iii) the role of galectins; and (iv) the activation of purinergic 2X7 receptor (P2X7R). Moreover, this review proposes the selective inhibition of galectins and the P2X7R as a potential pharmacological approach to prevent the progression of DR.

## 1. Diabetic Retinopathy (DR): An Overview

Diabetes is known as a chronic disease with multiple complications [1]. One of the most harmful and common complications is diabetic retinopathy (DR) [2,3]. This is a leading cause of blindness worldwide [4], with 103.1 million adults affected by DR in 2020 and a predicted 160.5 million affected in 2045 [5]. Moreover, DR individuals often develop a visual impairment, leading to a poor quality of life for the patient and impacting on the health care system in terms of direct and indirect costs. Indeed, in 2020, there were 28.5 million patients affected by vision-threatening DR, and it is estimated this number will be 44.8 million by 2045 [5].

Historically, DR has been considered as a microangiopathy, and is characterized by increased retinal vascular permeability and endothelial damage [2,6,7]. In particular, the hallmarks of DR onset include the thickening of the basement membrane, the loss of pericytes, the alteration of tight junctions and the endothelial barrier, the formation of microaneurysms and the uncontrolled proliferation of endothelial cells [8,9,10]. These events later translate into capillary occlusion that anticipates retinal ischemia, which is, in turn, associated with vascular endothelial growth factor (VEGF) overexpression and neovascularization [9,11].

Accordingly, DR can be clinically classified as either non-proliferative diabetic retinopathy (NPDR) or proliferative diabetic retinopathy (PDR) [8], which are usually diagnosed by fluorescein angiography (FA) or mydriatic fundus camera examinations [12]. However, other imaging methods such as optical coherence tomography (OCT), Doppler OCT, OCT angiography and retinal imaging of the fundus can be used [12]. While NPDR is the early stage of the disease (which can be further classified as mild, moderate and severe), PDR represents the advanced stage of retinopathy. NPDR is characterized by progressive changes in retinal capillary microcirculation, resulting in microaneurysms, blood–retinal barrier (BRB) breakdown and intraretinal exudates [13]. Specifically, mild NPDR is diagnosed by the presence of few microaneurysms; moderate NPDR is characterized by microaneurysms, venous beading or intraretinal hemorrhages; severe NPDR is defined by the presence of hemorrhages or microaneurysms or both, venous beading or prominent intraretinal microvascular abnormalities (IRMAs) [14]. In contrast, PDR is characterized by the presence of numerous ischemic retinal areas, VEGF overproduction and neoangiogenesis [15,16], and is diagnosed by neovascularization of the disc retina and iris or by tractional retinal detachment or vitreous hemorrhage [14]. Indeed, PDR can evolve into more severe stages such as diabetic macular edema (DME), neovascular glaucoma and, in rare cases, retinal detachment [9,13,15,17].

While the glycemic, lipidic and blood pressure controls are needed for DR primary prevention, the current surgical and pharmacological options for DR management include pan-retinal laser photocoagulation in severe NPDR and PDR patients, focal retinal laser photocoagulation in patients with diabetic DME, anti-VEGF intravitreal injections in PDR and DME patients, and steroid intravitreal injections for non-responder DR patients or naïve patients when appropriate [18]. In particular, the early use of laser photocoagulation or intravitreal injections of glucocorticoids or anti-angiogenics drugs has proven effective in preventing the onset of blindness [19], while anti-VEGF intravitreal injections are considered the actual gold standard for DR therapy. However, DR management is still challenging, with the 40% of inadequately treated NPDR cases evolving to PDR within 12 months [13,20]. Therefore, several studies have been performed to identify new molecular pathways or circulating biomarkers as new potential pharmacological tools in DR prevention and/or treatment. Among these, an important role as predictive and therapeutic targets in DR progression has been proposed for melanocortin receptors, retinol binding protein 3 (RBP3), angiopoietin-like 3 (ANGPTL3), microRNAs, extracellular vesicles, metabolites as 12-hydroxyeicosatetraenoic acid and 2-piperidone, along with several mediators involved in the resolution of inflammation [21,22,23,24,25,26,27].

Currently, intraretinal inflammation and neurodegeneration have emerged as key processes in DR progression [16,28]. Indeed, along with microvascular damage, inflammatory and neurodegenerative processes contribute to alterations in the “retinal neurovascular unit”, a functional unit composed of endothelial cells, glial cells and neurons [29]. In this regard, early retinal neurovascular alterations have been recently associated with specific serum microglial biomarkers such as ionized calcium-binding adapter molecule 1 (Iba-1), glucose transporter 5 (GLUT5), and translocator protein (TSPO) [27].

In this review, we focus on the importance of endoplasmic reticulum stress (ERS) and the consequent activation of the nucleotide-binding oligomerization domain-, leucine-rich repeat- and pyrin domain-containing 3 (NLRP3) inflammasome during retinal neuroinflammation. In particular, ERS modulation by specific proteins binding beta-galactoside residues on glycated proteins known as galectins is detailed. The galectin family consists of 15 identified members, all of which contain conserved carbohydrate-recognition domains (CRDs) of about 130 amino acids [30]. Based on the CRD organization of the polypeptide monomer, the galectins have been classified into three types: proto-type, chimera-type, and tandem-repeat-type [31]. The prototypical galectins have one CRD (galectin-1, -2, -5, -7, -10, -11, -13, -14 and -15); the tandem-repeat-type galectins contain two homologous CRDs in a single polypeptide chain, separated by a linker of up to 70 amino acids (galectin-4, -6, -8, -9 and -12); galectin-3 contains a non-lectin N-terminal region (about 120 amino acids) connected to a CRD [32]. Outside the cell, galectins bind to cell-surface and extracellular matrix glycans; however, galectins are also detectable in the cytosol and nucleus. They may influence cellular signaling pathways, such as cell migration, autophagy, immune response, and inflammation [32].

Moreover, the importance of purinergic 2X7 receptor (P2X7R) in NLRP3 activation underlying neuroinflammation is analyzed, along with the options for their selective inhibition in several clinical settings. Specifically, the state of the art on galectins and P2X7R inhibitors in DR is described, along with their potential use as pharmacological tools to prevent DR progression.

## 2. The Role of Neuroinflammation in DR

The “retinal neurovascular unit” includes specific retinal glia elements, such as microglial cells and macroglia (Müller cells and astrocytes) [28,33,34]. These express marked ERS [35,36,37] and subsequent activation of NLRP3 inflammasome [38], as a consequence of advanced glycation end products (AGEs) and reactive oxygen species (ROS) formation, strongly induced by chronic hyperglycemia [39]. In this regard, increases in many pro-inflammatory cytokines and chemokines, such as interleukin 1 beta (IL-1β), interleukin 6 (IL-6), interleukin 8 (IL-8), tumor necrosis factor alpha (TNF-*α*) and monocyte chemoattractant protein-1 (MCP-1), have been found in serum and in ocular samples (vitreous and aqueous humor) of DR patients. The accumulation of these cytokines is mainly mediated by cells from the macroglia and microglia [40].

Microglial cells represent the first defense line in neural retina [41,42,43]. Specifically, the retinal inner layers are characterized by the presence of ramified microglial cells [44,45] controlling the regulation of retinal growth, blood vessel formation and the retinal endothelial cell–glia–neuron interactions [46,47,48,49,50]. When activated by hyperglycemia, retinal hypoxia, ischemia and ER stress [51,52,53,54], these cells are classified as M1 macrophages and release pro-inflammatory mediators such as IL-6 and IL-12, TNF-*α* and interferon gamma (IFN-*γ*), known inducers of inflammation, apoptosis and neurotoxicity [55,56,57]. Microglial cells can be alternatively classified as M2 macrophages and release anti-inflammatory mediators, thus exerting anti-inflammatory effects and neuroprotection [42,55,58].

Retinal microglia polarization directly influences Müller cells and macroglia cells, spanning the entire retinal width and connecting neurons and vascular components [59,60]. In healthy retina, Müller cells contribute to the BRB integrity, modulate retinal blood flow and regulate the production of ions, neurotransmitters and metabolites that favor retinal blood vessel dilatation or constriction [61]. As a consequence of hyperglycemia-induced ER stress [35,39], Müller cell activation leads to the release of pro-inflammatory cytokines and chemokines [62] which favor leucocytes recruitment [63], a phenomenon termed gliosis. Moreover, Müller cell inflammatory actions across the retinal laminal structure seems to favor microglia attraction and adhesion by modulating retinal injury response [60].

Lastly, astrocytes are the most abundant central nervous system (CNS) macroglial cell type, forming the inner retinal BRB [64]. These are prevalent in the retinal ganglion cell layer (GCL) and nerve fiber layer (NFL) and modulate the neuronal metabolism, neurotransmission, and the neurorepair process [65,66,67]. Astrocytes can exert either pro- or anti-inflammatory actions, depending on the microenvironment in which they are located and the received signal [68].

## 3. Mechanisms of DR Neuroinflammation

### 3.1. Endoplasmic Reticulum (ER) Stress

In the diabetic retina, hyperglycemia leads to elevated ROS production by various mechanisms, including the reduction of antioxidant enzymes [69] and the activation of hypoxia-inducible transcription factor 1 (HIF-1) [70,71].

**Figure 1:**
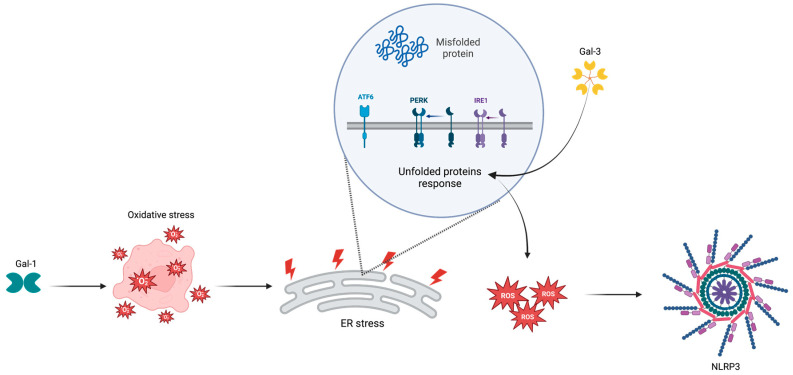
Involvement of galectins in ER stress. ATF6: activating transcription factor; ER: endoplasmic reticulum; Gal-1: galectin 1; Gal-3: galectin 3; IRE1: inositol-requiring kinase 1; NLRP3: nucleotide-binding domain, leucine-rich–containing family, pyrin domain–containing-3 inflammasome; PERK: protein kinase R-like endoplasmic reticulum kinase; ROS: reactive oxygen species. Arrow: increase. Created with BioRender.com, accessed on 2 May 2023.

During DR, the hyperglycemia-induced ER stress (ERS) affects all the components of the “neurovascular unit” and leads to the apoptosis of both vascular and neuronal cells [72,73]. In particular, the abnormal ERS causes the apoptosis of retinal pericytes [74] with BRB impairment and retinal neuroinflammation [75,76]. Among the ERS inducers, key roles are played by glycative stress and activation of the unfolded protein response (UPR) that are stimulated by AGEs and ROS formation, respectively [77]. Glycative stress is used to monitor the status of protein folding and ensure that only properly folded proteins are trafficked to the Golgi [78,79]. UPR is an adaptive signaling pathway which tends to enhance the ER capacity for protein folding and modification to restore an efficient protein-folding environment [77]. The main UPR actors are the inositol-requiring kinase 1 (IRE1), the protein kinase R (PKR)-like endoplasmic reticulum kinase (PERK) and the activating transcription factor 6 (ATF6) [77,80]. When the accumulation of protein aggregates exceeds the ER load capacity, the activation of UPR exacerbates ERS [81] (Figure 1).

ERS-induced microglial activation occurs during the different DR stages [27,47]. Indeed, activated microglial cells have been observed in the retinal plexiform layers (RPL) of NPDR patients and around the ischemic areas in PDR patients [82]. Notably, changes in M1/M2 microglia polarization can lead to visual loss by increasing the apoptosis of retinal neurons with consequent retinal NFL thinning [83].

Conversely, ERS-induced Müller cell activation seems to precede the DR vascular alterations [84], with NPDR patients showing Müller cell gliosis and swelling in the retinal inner nuclear layer (INL) and outer plexiform layer (OPL) [85], although PDR neuronal damage and DME cyst formation also characterize gliosis [86]. Lastly, retinal astrocytes are responsible for a sustained production of the pro-inflammatory IL-1β in response to hyperglycemia-induced ERS [87].

### 3.2. NLRP3 Inflammasome

ROS-induced ER stress leads to the activation of the NLRP3 inflammasome, a multiproteic complex found as monomer when inactive [88,89,90,91] (Figure 1). In particular, ERS exacerbation leads to activation of the NLRP3 inflammasome [92] through the binding of thioredoxin-interacting protein (TXNIP) to NLRP3 [93,94].

**Figure 2:**
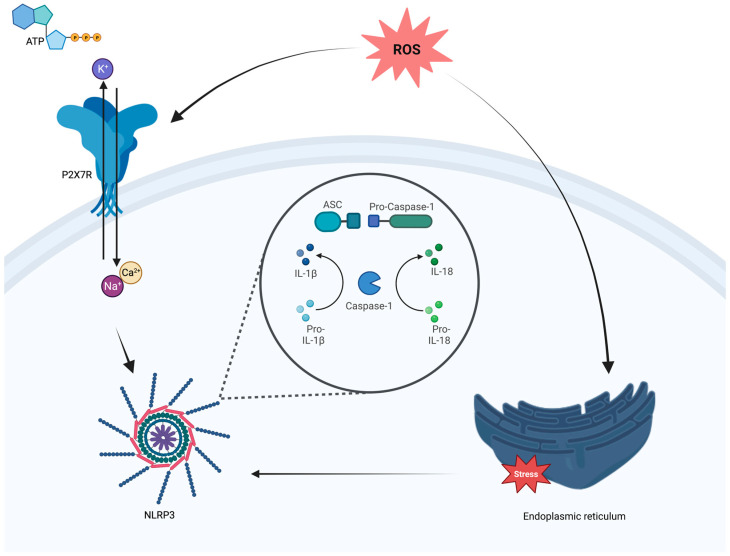
Involvement of P2X7R in NLRP3 activation. ASC: apoptosis-associated speck-like protein; ATP: adenosine triphosphate; Ca^2+^: Calcium; IL-1β: interleukin 1 beta; IL-18: interleukin 18; P2X7R: purinergic 2X7 receptor; ROS: reactive oxygen species; Na^+^: sodium; NLRP3: nucleotide-binding domain, leucine-rich-containing family, pyrin domain-containing-3 inflammasome; K^+^: potassium. Arrow: increase. Created with BioRender.com, accessed on 2 May 2023.

The NLRP3 complex consists of a sensor component (NLRP3 protein), an effector component (caspase-1) and, in some cases, an adapter protein (known as apoptosis-associated speck-like protein—ASC) linking the sensor and the effector [93,95,96]. The NLRP3 sensor is characterized by three different domains known as cytosolic pattern recognition receptors (PRRs): an amino-terminal pyrin domain (PYD), a central nucleotide-binding and oligomerization domain (NOD domain), and a C-terminal leucine-rich repeat (LRR) domain. Through these, NLRP3 is known to sense an extremely broad range of both exogenous and endogenous stimuli, known as pathogen-associated molecular patterns (PAMPs) and danger-associated molecular patterns (DAMPs), including changes in the ion gradient across the cell, cellular stress mechanisms such as higher ROS production after lysosomal rupture, mitochondrial stress and ERS in response to the accumulation of misfolded proteins [97,98,99,100]. When activated, the pyrin domain of NLRP3 interacts with the ASC pyrin domain to initiate inflammasome assembly [100,101] (Figure 2).

The pyrene domain connects to the NLRP3 pyrene domain through an oligomerization process; in addition, the adaptor protein ASC brings pro-caspase 1 monomers near to each other through the CARD domain by inducing a proximity-mediated caspase-1 autoactivation [101]. Active caspase-1 can induce the release of pro-inflammatory cytokines, such as IL-1β and interleukin 18 (IL-18), favoring both apoptosis and pyroptosis, a cell death process during which some pro-inflammatory cytokines are released to attract and activate immune cells [93,95,102,103]. Moreover, active caspase-1 is also necessary for the proteolytic cleavage of Gasdermin D (GSDMD) and the consequent release of the GSDMD N-terminal fragment, which is necessary to mediate pyroptosis [104].

Similarly, NLRP3 is pivotal in DR progression [89,91,105,106,107]. Indeed, retinal NLRP3 levels were increased in serum and vitreous samples from patients affected by PDR compared with NPDR patients [40,106,107].

NLRP3 activation is shown by retinal epithelial [108,109,110,111] and endothelial cells [112,113,114]. However, it is evident also in retinal neuronal layers, such as retinal ganglion cells (RGC) in mouse models of retinal degeneration and in photoreceptors from a genetic mouse DR model [115,116,117]. Moreover, NLRP3 levels were increased in retinal microglial cells exposed to high glucose [118] and in retinal macroglia from a mouse model of ocular hypertension [119].

## 4. New Actors in Neuroinflammation

### 4.1. Galectins and ERS

Overall, galectin dysregulation has been linked to different pathological conditions, such as fibrosis, heart disease, cancer, and diabetes [32]. In this regard, Gal-1 has been associated with type 2 diabetes [120], while Gal-3 has been correlated to both type 1 and type 2 diabetes [121,122,123], with a suggested role for this galectin in mediating the chronic inflammation underlying the progression from prediabetes to the diabetic stage [124]. Moreover, in diabetic patients, diabetic nephropathy, diabetic foot, diabetic microvascular complications, and diabetic cardiomyopathy have been all related to changes in Gal-3 serum levels [125]. In particular, Galectin 1 (Gal-1) and Galectin 3 (Gal-3) seem to initiate the inflammatory response by acting as chemotactic agents towards the inflammatory site for the neutrophils, facilitating their binding to the endothelium and their trafficking through the extracellular matrix [126].

Galectins, induced by AGEs and considered as receptors for AGEs (RAGE), have emerged as ERS regulators. For example, the elevation of Galectin 9 (Gal-9) has been linked to inflammatory processes in both type 1 and type 2 diabetes [127,128]. However, an additional protective role against ERS has been described for Gal-9 [129], along with its importance in the facilitation of NLRP3 autophagic degradation [130].

In addition, Gal-1 and Gal-3 have also emerged as ERS regulators (Figure 1). Specifically, Gal-1 is upregulated in hypoxic microenvironments [131], resulting in increased ROS production and activation of the nuclear factor kappa B (NF-κB) signaling pathway [132]. This protein is considered a key regulator of endothelial cells functions and shows potent proangiogenic properties [133,134].

Gal-3 is localized at the ER-mitochondria interface and regulates the UPR [135]. It is involved in several processes underlying retinopathies, such as oxidative stress, proliferation, phagocytosis, apoptosis, oxidative stress, and angiogenesis [136]. Interestingly, Gal-3 may favor adaptive UPR following ERS by acting as both a pro- and anti-apoptotic regulator [135]. Tian and colleagues demonstrated that Gal-3 is also implicated in the process of activating the NLRP3 inflammasome, discovering a direct link of the N-terminal domain of Gal-3 to the NLRP3 inflammasome [137]. Several studies have shown alterations in the concentration of galectins in the brain and blood of patients with neurodegenerative diseases compared with healthy subjects. In particular, Gal-1 was found in neurofilamentous lesions of patients affected by amyotrophic lateral sclerosis (ALS) [138], while patients with multiple sclerosis showed a higher concentration of Galectin 4 (Gal-4) in chronic lesions of the brain [139]. However, Gal-3 can be considered as an indicator of prognosis, mortality or remission in neurodegenerative diseases [140] since its expression was found to be increased in patients with ALS, Alzheimer’s and Parkinson’s diseases (AD and PD, respectively) [141].

This could be firstly due to the high affinity showed by galectins for β-galactosides [142,143], which are involved in neuroprotection and neuroinflammation [144,145]. Moreover, galectins are expressed by glial cells. In particular, Gal-1 expression was reported in astrocytes and Müller cells, participating in the protection from axonal damage as it mediates T-cell activation and differentiation, whereas Gal-3 expression was mainly observed in M1 microglial cells and was associated with microglial activation in cell damage, ischemia, and encephalitis [30,140,146]. Particularly astrocyte-derived Gal-1 seems to play a key role in the modulation of inflammation, phagocytosis, axon growth and gliosis after spinal cord injury [147,148]. It was also found to be important in the modulation of microglia polarization and neuromodulation in a multiple sclerosis model [149], as well as modulation of axonal degeneration in a transgenic mouse model of ALS [150]. Regarding Gal-3, in primary rat microglia and macroglia, Gal-3 exposure increases the expression of TNF-α, IL-1β, IL-6 and INF-γ [151]. Accordingly, in a mouse model of Huntington’s disease, Gal-3 increased the microglial expression of IL-1β [152]. Moreover, the suppression of Gal-3 in AD mice improved their cognitive performance, reducing amyloid plaques [153], as well as cognitive impairment, neuroinflammation and oxidative stress associated with diabetes in rats caused by modified citrus pectin (MCP) [154].

### 4.2. Purinergic 2X7 Receptor (P2X7R) and NLRP3 Inflammasome

P2X7R is a trimeric ion channel that belongs to the P2X family of ionotropic receptors preferably permeable to sodium, potassium and calcium, which are exclusively triggered by extracellular adenosine triphosphate (ATP) [96]. P2X receptors are widespread in different tissues and exert various functions: in some smooth muscle cells, activated P2X receptors mediate depolarization and contraction; in the CNS, activated P2X receptors allow calcium to enter neurons, leading to slower neuromodulatory responses; and in the cells of the immune system, activated P2X receptors trigger the release of pro-inflammatory cytokines [155].

P2X7R presents five domains: one extracellular domain; two transmembrane domains (classified as transmembrane 1 and 2); and two intracellular domains (N- and C-terminus), which form homotrimeric receptors after their activation [156,157]. Its signaling is important for regulating both the innate and adaptive immune response [156] and also inflammatory processes [96].

P2X7R is highly expressed in different cell types and tissues, such as retinal neural cells and the retinal vasculature [158], but also in immune cells [159]. In particular, in the human retina, P2X7R is localized in INL, OPL and GCL and is expressed by human retinal Müller cells, the native retinal pigment epithelium (RPE) and adult retinal pigment epithelial cell line-19 (ARPE-19) [160].

P2X7R has a key role in linking inflammation with purinergic signaling: this receptor and the NLRP3 inflammasome interact at distinct cytoplasmic sites, where changes in P2X7R-dependent ion concentrations (specifically a K+ efflux) occur [156,161,162], probably mediated by the cytoplasmic kinase never-in-mitosis A- related kinases (NEK) [156] (Figure 2). In particular, during NLRP3 inflammasome activation, the K+ efflux seems to be mediated by P2X7R following an increase in extracellular ATP [163]. This allows the release of pro-inflammatory cytokines associated with NLRP3 inflammasome activation [156]. In this regard, P2X7R is upregulated [162] in pathological conditions as a consequence of increased extracellular microglial ATP concentration [164]. This is indicative of the pro-inflammatory M1 phenotype microglia activation [164]. P2X7R is widely expressed in the CNS regions and is associated with neuroinflammation and neurodegeneration [164,165,166]. Therefore, P2X7R has been extensively investigated in order to develop small molecules that could act as potent blockers of the receptor [155].

Indeed, P2X7R activation in glial cells overall results in the release of the pro-inflammatory cytokines, thereby triggering or potentiating neuroinflammation [167]. This contributes to neurodegeneration by inducing microglia-mediated neuronal death [168], glutamate-mediated excitotoxicity and NLRP3 inflammasome activation, with the consequent release of IL-1β and IL-18 [96,164,169].

Several studies evidenced that P2X7R expression is upregulated in the activated microglia of AD patients and is concentrated in amyloid plaques [170,171]. Similarly, P2X7R was upregulated in the hippocampus of an AD animal model [172,173]. Furthermore, preclinical evidence suggests that P2X7R may have a role in the pathogenesis of Huntington’s disease [174]. P2X7R also has a possible involvement in PD by mediating activation of the NLRP3 inflammasome [175]. This aspect is under investigation in an observational prospective study at the University of Pisa (Italy). This study is evaluating changes in P2X7R levels and their association with changes in NLRP3 inflammasome levels in patients with newly diagnosed PD or AD receiving routine treatment in comparison with an age- and gender-matched group [176]. P2X7R was also recently identified as a key contributor to cognitive impairment in a mouse model of migraine; activation of the NLRP3 inflammasome and P2X7R upregulation led to gliosis, neuronal loss and neuroinflammation [177]. Astrogliosis related to P2X7R has also been reported in a rat model of autoimmune encephalomyelitis [178].

## 5. Inhibition of Galectins and P2X7R and Its Potential Therapeutic Application for DR Neuroinflammation

It is well known that galectins and P2X7R are two key mediators in DR pathology and progression [84,157,179,180,181,182,183,184,185,186,187,188,189]. Therefore, these neuroinflammatory actors represent two potential pharmacological targets to prevent the onset of DR by their selective inhibition.

In this regard, different compounds inhibiting galectins have been evaluated in clinical trials. In particular, although Gal-1 inhibitor OTX0008 has been tested in patients with advanced solid tumors [190], Gal-3 inhibition has gained a wider application in several ongoing clinical trials. Indeed, both the Gal-3 inhibitors GR-MD-02 and GB1211 are in evaluation for liver fibrosis in non-alcoholic steatohepatitis (NASH) [143,191,192]. Administration of GB1211 alone has also been investigated for hepatic impairment [193], and its combination with atezolizumab (a monoclonal antibody targeting the Programmed Death Ligand-1) has been considered in non-small cell lung cancer patients [194]. Furthermore, the co-administration of GR-MD-02 with ipilimumab (a monoclonal antibody targeting the Cytotoxic T-Lymphocyte Antigen 4) or pembrolizumab (a monoclonal human antibody targeting the Programmed Cell Death protein 1) is undergoing evaluation in patients with metastatic melanoma [195] and in patients with advanced melanoma, non-small cell lung cancer and head and neck squamous cell cancer, respectively [196,197].

Gal-3 inhibition by GB0139 and TD139 compounds is under evaluation in idiopathic pulmonary fibrosis (IPF) [143,198,199], while MCP as a Gal-3 inhibitor has been considered for both hypertension and osteoarthritis [200,201].

Regarding P2X7R, different compounds have been tested on healthy volunteers to assess their safety and tolerability, bioavailability, pharmacokinetics, and pharmacodynamics as P2X7R antagonists. These include GSK1482160 [202], AZD9056 [203,204], and ce-224,535 [205,206]. In particular, ce-224,535 and AZD9056 have both been in evaluation for patients with rheumatoid arthritis (RA) [207,208], with ce-224,535 considered also for patients with knee osteoarthritis pain [209].

To date, none of these inhibitors has been considered for DR patients. However, several pre-clinical studies evidenced the potential role of galectin and P2X7R inhibition strategies in modulating diabetic retinal damage [179,181,183,184].

### 5.1. Galectin Inhibition in DR

Gal-1 and Gal-3 have been recently associated with the insurgence and progression of DR pathology (Table 1).

In particular, Gal-1 levels were increased in the vitreous fluid and epiretinal fibrovascular membrane of PDR patients compared with non-diabetic controls [179]. This was probably due to the higher Gal-1 secretion induced in retinal Müller cells and astrocytes by hyperglycemic conditions [136]. The upregulation of Gal-1 protein levels in vitreous samples rose substantially with DR progression, being present from the pre-ischemic inflammatory stage [186]. Increased Gal-1 plasma levels were also detected in PDR patients compared with non-diabetic controls, along with a Gal-1 correlation with AGEs and IL-1β [185]. In line with this evidence, Gal-1 was also found to be upregulated in neovascular ocular tissues surgically excised from PDR patients, where it exhibited a co-localization with Vascular Endothelial Growth Factor Receptor 2 (VEGFR2) [186]. While in this study, Gal-1 exhibited no correlation with VEGFA, in a mouse model of oxygen induced-retinopathy (OIR), Gal-1 was shown to mediate vascular alterations concomitantly to VEGF up-regulation [187].

Gal-1 also seems to be involved in the regulation of the RPE that forms the BRB. Indeed, ARPE-19 cells exposed to high glucose exhibited high Gal-1 levels, which was associated with epithelial fibrosis and epithelial-mesenchymal transition [181]. Both processes were reduced by selective Gal-1 blocking with OTX008 [181].

Although Gal-1 has been associated with neuronal alterations in a mouse OIR model, and particularly with neuroglial injuries at post-natal day 26 [187], retinal neuroinflammatory actions have been specifically shown for Gal-3, whose pro-inflammatory role in diabetic optic neuropathy occurs through ROS-induced ERS [84]. Accordingly, within the neuronal retina, Gal-3 knockout resulted in reduced microglial activation and led to better preservation of RGC, nerve fibers, axons and cell bodies in diabetic mice [180]. This evidence was recently confirmed by Mendonça and colleagues [188], who showed that Gal-3 knockout in diabetic mice attenuated neuroinflammation in the retina and optic nerve. This effect was exerted by reducing the activation of retinal microglia and macroglia, and by increasing the number of myelinated fibers. Gal-3 knockout has also been associated with AGEs and VEGF reduction, along with the amelioration of BRB dysfunction, during short-term diabetes in mice [189].

### 5.2. P2X7R Inhibition in DR

Recent evidence suggests a role of the P2X7 receptor in controlling the BRB function and integrity [210,211]. Once P2X7R is activated, it mediates inflammatory vascular reactions induced by cytokines which degrade the BRB integrity and lead to retinal vascular occlusion and ischemia [182,210,212]. Moreover, P2X7R seems to mediate the accumulation of microglia and macrophages in the subretina [213].

A specific role for the P2X7R-NLRP3 inflammasome pathway in retinal endothelial inflammation and pericyte loss during the NPDR stage has been also described [182,183,184] (Table 2). Moreover, P2X7R stimulation or overexpression positively regulates VEGF secretion and accumulation, thus promoting DR neo-angiogenesis [157].

Therefore, a P2X7R inhibition strategy could represent a useful therapeutic tool to manage the early phase of DR. In this regard, the P2X7R selective inhibitor A74003 reduced the apoptosis of mice retinal endothelial cells (mRECs) stimulated with high glucose and lipopolysaccharide (LPS) [183]. Furthermore, Platania and colleagues analyzed the possible anti-inflammatory role of the selective P2X7R antagonist JNJ47965567 in diabetic human retinal pericytes [184]. While high glucose levels induced pericyte cell damage and a significant release of IL-1β, the treatment with JNJ47965567 decreased the IL-1β release by blocking P2X7R and consequently, NLRP3 inflammasome activation [184].

## 6. Conclusions

**Figure 3:**
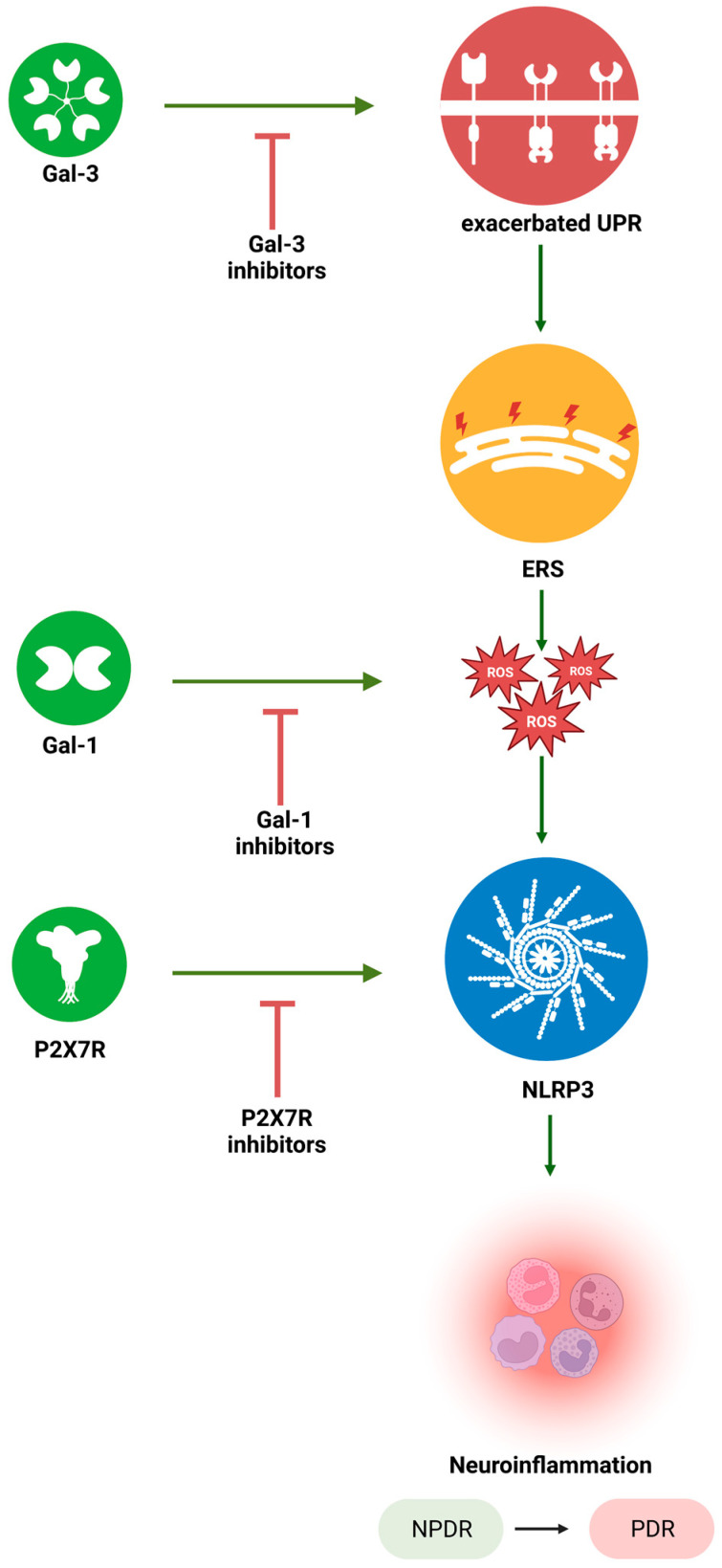
Diagram summarizing galectins, P2X7R and their inhibition of the ERS-NLRP3 inflammasome. ERS: endoplasmic reticulum stress; Gal-1: galectin 1; Gal-3: galectin 3; P2X7R: P2X7 receptor; NLRP3: nucleotide-binding domain, leucine-rich–containing family, pyrin domain–containing-3 inflammasome; NPDR: non-proliferative diabetic retinopathy; PDR: proliferative diabetic retinopathy; ROS: reactive oxygen species; UPR: unfolded protein response. Black arrow: progression; green arrow: increase; red arrow: decrease. Created with BioRender.com, accessed on 2 May 2023.

Due to the role of galectins in ERS modulation and the role of P2X7R in NLRP3 inflammasome activation, both these mediators could represent two new potential targets, whose specific inhibition could help counteract the inflammatory process underlying DR progression, although evidence for a specific molecular relation and interlink between them is not available yet (Figure 3).

## Figures and Tables

**Table 1:** Galectins in DR studies.

Study	Main Results	Treatments	Reference
Clinical Vitreous fluid samples from 36 patients with PDR (20 patients undergoing vitrectomy as controls)	VEGF and Gal-1 levels significantly higher in PDR patients compared with controls	-	[179]
Clinical Plasma samples from 20 PDR cases (20 non-diabetic, idiopathic macular diseases as controls)	Plasma levels of Gal-1, AGEs and IL-1β significantly increased in PDR patients	-	[185]
Clinical 23 vitreous fluids aspirated from PDR (non-diabetic control eyes with idiopathic ERM and MH) Neovascular ocular tissues surgically excised from PDR patients (non-diabetic tissues as controls)	Gal-1 levels were significantly elevated in the vitreous fluids of PDR eyes compared with controls Gal-1 upregulated in PDR tissues and co-localized with VEGFR2	-	[186]
In vivo STZ-Sprague Dawley rats (non-diabetic rats as controls) STZ:55 mg/kg (single dose, i.v. injection) Vehicle: 10 mM sodium citrate buffer (single dose i.v. injection)	Retinal Gal-1 levels increased in diabetic rats compared with controls	-	[179]
In vivo Gal-3 knockout C57/BL6 mice (wild-type mice as controls)	Gal-3 knockout mice exhibited less activated inflammatory cells within the optic nerve after crush	-	[180]
In vivo Diabetic Gal-3 knockout C57/BL6 mice (wild-type diabetic mice as controls) STZ:160 mg/kg (single dose, i.p. injection) Vehicle: sodium citrate buffer (single dose i.p. injection)	Gal-3 knockout reduced RGC apoptosis, Iba-1 and GFAP in the distal optic nerve in diabetic mice; moreover, it prevented the loss of myelinated fibers	-	[188]
In vivo Diabetic Gal-3 knockout C57/BL6 mice (wild-type diabetic mice as controls) STZ:165 mg/kg (single dose, i.p. injection) Vehicle: sodium citrate buffer (single dose i.p. injection)	Gal-3 knockout reduced AGEs, VEGF and BRB breakdown in diabetic mice	-	[189]
In vitro Hypoxic (CoCl_2_) human retinal Müller glial cells (non-hypoxic cells as controls) CoCl_2_: 300 μM for 24 h	OTX008 attenuated the upregulation of Gal-1, VEGF and NF-*κ*B in hypoxic retinal Müller cells	Gal-1 selective inhibitor OTX008 OTX008: 10 μM for 24 h	[179]
In vitro ARPE-19 cells exposed to HG (NG cells as controls) HG: 35 mM NG: 5 mM	OTX008 induced a significant increment in cell viability; while Gal-1 protein, ROS and TGF-β1 levels were reduced after OTX008	Gal-1 selective inhibitor OTX008 (2.5–5–10 µM)	[181]

Abbreviations. AGEs: advanced glycation end products; ARPE-19: adult retinal pigment epithelial cell line-19; BRB: blood-retinal barrier; CoCl_2_: cobalt chloride; ERM: epiretinal membrane; Gal-1: galectin 1; Gal-3: galectin 3; GFAP: glial fibrillary acidic protein as activated macroglia marker; h: hours; HG: high glucose; Iba-1: ionized calcium-binding adapter molecule 1; IL-1β: interleukin 1 beta; i.p.: intraperitoneal; i.v.: intravenous; MH: macular hole; NF-kB: nuclear factor kappa-light-chain-enhancer of activated B cells; NG: normal glucose; OTX008: calixarene 0118; PDR: proliferative diabetic retinopathy; RGC: retinal ganglion cells; ROS: reactive oxygen species; STZ: streptozotocin; TGF-β1: transforming growth factor beta 1; VEGF: vascular-endothelial growth factor; VEGFR2: Vascular Endothelial Growth Factor Receptor 2.

**Table 2:** P2X7R in DR studies.

Study	Treatment	Main Results	References
In vivo STZ-C57BL/6J mice (non-diabetic mice as controls) STZ:50 mg/kg (daily, i.p. injection) for 5 days Vehicle: 0.1% mol/L citrate buffer (daily, i.p. injection) for 5 days	P2X7R selective inhibitor A74003 (100 µg/kg/d); NLRP3 inflammasome selective inhibitor MCC950 (100 µg/kg/d) A74003: 100 µg/kg/d, i.p. injection every alternate day from week 9 to week 12 MCC950: 100 µg/kg/d, i.p. injection daily for the first 3 days and then every alternate day from week 9 to week 12	P2X7R mRNA Significantly higher in the retinal tissues of STZ-mice; A74003 and MCC950 reduced retinal inflammation and apoptosis in STZ-mice	[183]
In vitro Pericyte-containing retinal microvessels from STZ-Long Evans rats (non-diabetic rats as controls) STZ: 75 mg/kg (single dose, i.p. injection) Vehicle: 0.8 mL (single dose, citrate buffer i.p. injection)	P2X7R agonist BzATP BzATP: 100 µM for 24 h	BzATP (100 µM) increased the apoptosis of pericyte-containing retinal microvessels from STZ-rats	[182]
In vitro mRECs exposed to HG, alone or in combination with LPS, (NG cells as controls) HG: 50 mM for 24 and 48 h LPS: 20 ng/mL for 48 h NG: 5.5 mM for 24 and 48 h	P2X7R selective inhibitor A74003; NLRP3 inflammasome selective inhibitor MCC950 A74003: 100 µmol/L 6 h before HG or HG + LPS, until 72 h MCC950: 100 µmol/L, 6 h before HG or HG + LPS, until 72 h	A74003 reduced apoptosis and pyroptosis in mRECS exposed to HG combined with LPS	[183]
In vitro Human retinal pericytes exposed to HG (NG cells as controls) HG: 25 mM for 48 h NG: 5 mM for 48 h	P2X7R agonist BzATP; P2X7R novel antagonist JNJ47965567 BzATP: 100 µM for 48 h JNJ47965567: 10–100 nM for 48 h	BzATP induced human retinal pericyte cell death and increased the pro-inflammatory cytokines levels; JNJ47965567 Protected pericytes viability and reduced pro-inflammatory cytokines	[184]

Abbreviations: BzATP: benzoylbenzoyl-adenosine triphosphate; h: hours; HG: high glucose; i.p.: intraperitoneal; LPS: lipopolysaccharide; mRECs: mice retinal endothelial cells; mRNA: messenger ribonucleic acid; NG: normal glucose; NLRP3: nucleotide-binding domain, leucine-rich–containing family, pyrin domain–containing-3 inflammasome; P2X7R: purinergic 2x7 receptors; STZ: streptozotocin.

## Data Availability

Not applicable.
